# Causes of Childhood Cancer: A Literature Review (2014–2021)—Part 3: Environmental and Occupational Factors

**DOI:** 10.3390/cancers17213516

**Published:** 2025-10-31

**Authors:** Rebecca T. Emeny, Mary E. Butow, Linda Titus, Angela M. Ricci, Pamela J. Bagley, Heather B. Blunt, Alexandra Morgan, Jennifer A. Alford-Teaster, Raymond R. Walston, Judy R. Rees

**Affiliations:** 1 Department of Internal Medicine, Division of Molecular Medicine, UNM Comprehensive Cancer Center, Cancer Control & Population Sciences Research Program, University of New Mexico Health Sciences, Albuquerque, NM 87131, USA; 2 New Hampshire Department of Health and Human Services, Concord, NH 03302, USA; 3 Department of Pediatrics, Geisel School of Medicine at Dartmouth, Dartmouth Cancer Center, Hanover, NH 03755, USA; 4 Department of Pediatrics, Division of Pediatric Hematology-Oncology, Dartmouth Health Children’s, Lebanon, NH 03756, USA; 5 Biomedical Libraries, Geisel School of Medicine at Dartmouth, Hanover, NH 03755, USA; 6 Department of Obstetrics and Gynecology, Dartmouth Health, Lebanon, NH 03756, USA; 7 New London Hospital Association, New London, NH 03257, USA; 8 Department of Pediatric Hematology Oncology, Children’s Hospital Colorado, Aurora, CO 80045, USA; 9 Department of Epidemiology, Geisel School of Medicine at Dartmouth, Dartmouth Cancer Center, Hanover, NH 03755, USA; 10 Public Health Institute, Cancer Registry of Greater California, Sacramento, CA 95825, USA

**Keywords:** pediatric cancer, epidemiology, etiology, environmental risk factors, occupational risk factors

## Abstract

**Simple Summary:**

Childhood cancer is a significant public health concern with increasing rates over decades suggesting that environmental factors may contribute to this trend. We reviewed studies published between 2014 and 2021 that examined childhood exposure to air pollution and radiation, as well as parental environmental and occupational exposures before conception or during pregnancy, in relation to childhood cancer. Strong evidence was found for associations between leukemia and exposures to traffic pollution, indoor paint, residential pesticides, and occupational/nonoccupational benzene, and between brain cancer and exposures to residential pesticides and occupational agricultural pesticides. There was mixed evidence of associations between leukemia and electromagnetic fields (EMFs), and ionizing radiation from radon or nuclear power plants. This review consolidates knowledge of environmental factors associated with childhood cancer to inform targeted prevention strategies.

**Abstract:**

Purpose: To achieve a better understanding of the environmental factors that contribute to childhood cancers, so as to inform future prevention efforts. Methods: We conducted a comprehensive review of epidemiological studies on environmental risk factors and childhood cancer, which was published between January 2014 and March 2021. Potential exposure sources presented in this review include air pollution, radiation, and parental occupational exposures. We considered exposures during childhood and parental exposures occurring before the child’s conception and during pregnancy in relation to all types of childhood cancer. Results: Aggregated evidence is strong for associations between leukemia and parental/child exposure to traffic pollution, indoor paints, residential pesticides, and parental occupational/nonoccupational exposure to benzene. Evidence is also strong for associations between brain cancer and residential pesticides and parental occupational exposure to agricultural pesticides. Evidence of associations between leukemia and ionizing radiation from radon and nuclear power plants remains mixed, as does evidence of a link between electromagnetic fields (EMFs) and childhood leukemia. Conclusions: Clear associations have been demonstrated between childhood cancer and environmental factors, including parent/child exposure to traffic pollution, occupational/nonoccupational benzene, indoor paints, residential pesticides, and parental occupational exposure to agricultural pesticides. These associations can be used to inform further study of interventions and public health campaigns to reduce risk.

## 1. Introduction

This manuscript is the third of a three-part series summarizing the literature published between 1 January 2014 and 17 March 2021 on the causes of pediatric cancer (Table 1).

## 2. Materials and Methods

The methods for this literature review, with a detailed search strategy, were described previously [1]. Briefly, we identified primary research studies and reviews in Ovid Medline and Scopus from 1 January 2014 to 17 March 2021. We screened titles and abstracts of 3116 studies and 746 reviews (the combined results of our searches with duplicates removed). From the results of title/abstract screening, we selected 520 studies and 462 reviews for full-text screening using a team-based approach, summarizing the results by topic (Table 2) and discussing the weight of evidence for each. Except for large or influential studies, we generally omitted individual studies that contributed data to the meta or pooled analyses reviewed here. Here, we summarize peer-reviewed, published studies assessing outdoor and indoor sources of pollution and occupational exposures in relation to childhood cancers. Methods for the systematic search of the literature, including inclusion criteria, are detailed in the Methods section and Supplementary Materials of Ricci et al. 2024 [1]. Briefly, we searched for epidemiologic studies focused on cancer, including pediatric subtypes, and a variety of potential carcinogens.

In this paper, Section 3 addresses air quality categorized by outdoor (Section 3.1) and indoor (Section 3.2) sources of exposure. Section 4 reviews studies on radiation exposures from outdoor (Section 4.1) and indoor (Section 4.2) sources. Studies of parental exposures to carcinogens at work are covered under occupational exposures in Section 5, including benzene (Section 5.1), miscellaneous and other (Section 5.2), agricultural animals (Section 5.3), and agricultural pesticides (Section 5.4). Systematic reviews and meta-analyses on each topic are summarized with observed risk estimates presented as odds ratios (ORs) or relative risks (RRs) and 95% confidence intervals (CIs). A more general synthesis of other individual studies not included in the previously cited work is presented at the end of each section. We describe associations as having strong evidence where meta-analyses or multiple well-designed studies support an association. We use the term mixed evidence when associations differ across reports and are seen in <3 individual studies. We categorize evidence as weak when studies are sparse or findings are based largely on ecological study designs.

Children’s environmental exposures may be measured from birth until the time of diagnosis or may be inferred by exposures to the parent(s) before the child’s conception or during pregnancy. A few studies have directly measured components of air pollution, but most investigations have used indirect measures or proxy variables to indicate exposure.

## 3. Air Quality

### 3.1. Outdoor Exposures

Exposures to outdoor air pollution include vehicular traffic emissions as well as industrial or agricultural sources of particles, dusts, fibers, fumes, and specific chemical exposures. Outdoor sources of ionizing radiation exposure are reviewed in Section 4.1.

Studies of outdoor pollution are dominated by exposure to the chemical benzene, which is classified by IARC as Group 1 “carcinogenic to humans” [18]. Although benzene can contaminate water, ambient air accounts for nearly all benzene exposure; it enters the air through combustion processes (e.g., cigarette smoke, motor vehicle emissions, forest fires, and volcanoes) and evaporation of petrochemicals. Cigarette smoke has high exposure levels and accounts for nearly all benzene exposure among smokers. However, on a population-basis, most environmental benzene exposure is due to traffic-related pollution; consequently, benzene exposure is often measured indirectly through residential distance from dense traffic, major roads, and/or gasoline filling stations. Measures of traffic-related pollution also include direct assessment of specific toxins using monitoring devices that detect particulate matter (PM) of various sizes (e.g., 2.5 microns), nitrogen dioxide (NO_2_), and benzene.

Evidence for the association between traffic-related pollution and childhood leukemia was examined in seven meta-analyses published from 2014 to 2019 [4,5,6,7,8,9,10]. A moderate and statistically significant association between children’s residential proximity to traffic/traffic density and leukemia was observed in a meta-analysis of seven case–control studies (odds ratio [OR], 1.53; 95% confidence interval 1.12–2.10), but no association was found with prenatal exposure (four studies, OR 0.92; 0.78–1.09) [5]. Two other meta-analyses found either no association (n = 11 case–control studies with high heterogeneity I^2^ = 63%) between density of traffic and childhood leukemia [4], or only suggestive evidence (n = 21 case–control studies) for a role of traffic indicators, benzene, and NO_2_ exposures in relation to childhood leukemia [7], but neither of these meta-analyses considered the timing of exposure in subgroup analyses. The studies included in both of these meta-analyses were covered by a larger meta-analysis of twenty-six case–control and three cohort studies that also found limited evidence of an overall association between traffic pollution and childhood leukemia, but noted a modestly elevated risk with benzene exposure when comparing highest to lowest exposure (seven studies, RR 1.27; 1.03–1.56); additionally an association with AML was observed in subgroup analyses (five studies, RR 1.84; 1.31–2.59), particularly in children diagnosed before the age of six (two studies, OR 3.21; 1.39–7.42) [8]. A meta-analysis of three case–control studies showed a link between residential proximity to gasoline stations (either from conception or pregnancy to diagnosis or at the time of diagnosis) and childhood leukemia (RR 2.42; 1.51–3.89) [10]. Another meta-analysis from the same group comprising studies of traffic-related exposure in Europe (n = 9) and the U.S. (n = 4) produced a summary RR of 1.48 (12 studies, 1.10–1.99) and showed a stronger association between traffic pollution (considering exposure at birth or at diagnosis) and leukemia for AML (four studies, RR 2.07; 1.34–3.20) than for ALL (seven studies, RR 1.49; 1.07–2.08) [6]. The same meta-analysis noted somewhat stronger associations between traffic measures and leukemia in the U.S. (four studies, 2.02; 1.35–3.00) than in Europe (eight studies, 1.35; 1.09–1.68). In contrast, another review and meta-analysis found significant associations between traffic-related air pollutants and childhood leukemia in studies conducted in Europe (six studies, OR 1.56; 1.08–2.25) but not in North America (five studies, OR 1.08; 0.88–1.33) [9]. The study also noted higher associations with postnatal than prenatal exposures.

Other registry-based observational studies conducted in Texas have shown higher risk of non-Hodgkin lymphoma and central nervous system (CNS) cancers in children (age < 15) in relation to air concentrations of traffic pollutants including 1,3-butadiene and diesel particulate matter [41]. In Denmark, a registry-based case–control study used sophisticated models with adjusted geospatial air pollution risk-prediction to estimate levels of air pollution over time within geocoded addresses and found an association between non-Hodgkin lymphoma diagnosed before age 20 and higher concentrations of fine atmospheric particulate matter PM_2.5_ and black carbon, which is a subcomponent of PM_2.5_ [42].

Parental traffic pollution exposure during pre-conception and pregnancy timeframes has been associated with childhood cancer risk. For example, population-based case–control studies from Texas have shown higher risk of central nervous system (CNS) tumors associated with maternal residence in areas of high (versus low) roadway density [43], as well as a higher risk of embryonal cancers (including neuroblastoma, retinoblastoma, hepatoblastoma, and Wilms’ tumor) in children (age < 5) associated with maternal residential proximity to major roadways [44]. However, another population-based case–control study showed no association between maternal proximity to roadways and childhood leukemia [45].

Studies using data from air pollution monitors and the California Cancer Registry showed associations between Wilms’ tumor in young children (age < 6) and maternal residential exposure to formaldehyde or acetaldehyde during the third trimester of pregnancy [46]. Additionally, the risk of germ cell tumors and particularly teratomas (diagnosed before age 6) was associated with prenatal exposure to the solvent dichloromethane, which is classified by IARC as a probable carcinogen (Group 2A) [14,47]. An extensive assessment of traffic-related air toxins associated with germ cell tumors in children (age < 6) demonstrated increased risk of prenatal exposure to 1,3-butadiene and meta/para-xylene during the second trimester of pregnancy [48].

A large population-based cohort study in Ontario assessed cancer risk in children (age < 6) in relation to the mother’s pregnancy exposure to measured air toxins. After adjustment for PM_2.5_, NO_2_, and other factors, the results showed a 13% higher risk of overall pediatric cancer incidence per 10,000/cm^3^ increments in exposure to ambient ultrafine particle concentration during the first trimester of pregnancy [49]. In the prospective Norwegian Mother and Child Cohort Study, self-reported maternal exposure to gasoline or exhaust as a proxy measurement of benzene prior to or during pregnancy was associated with increased risk of childhood (age < 14) leukemia [50]. The nationwide Australian Study of Childhood Brain Tumors reported a higher risk of brain tumors in children (age < 15) associated with paternal (but not maternal) exposure to vehicle fuel (refueling four or more times per month) in the year before conception [51]. The greatest risk was associated with refueling six or more times per month. Associations were seen regardless of the type of vehicular fuel assessed.

Although findings for traffic pollution are relatively consistent, variations in study results may arise from differences in indirect measures of traffic density. Indirect measures may include parental car ownership, number of cars, density of traffic within a set distance, distance from roadways, or distance from facilities that service vehicles. In addition, emissions vary according to type of fuel (leaded or unleaded gasoline, diesel). Study findings may also reflect seasonal, geographic, or cultural differences related to the amount of time children are outdoors. Finally, observed associations may be influenced by the completeness of cancer ascertainment.

Industrial facilities also contribute to outdoor sources of pollution. Several population-based case–control studies using data from the Spanish Registry of Childhood Cancer from 1996 to 2011 assessed numerous industry-related carcinogens (IARC Groups 1–2B), including production/processes involving metals, pesticides, plasticizers, waste, solvents, and several chemicals, including polycyclic aromatic chemicals, non-halogenated phenolic chemicals, volatile organic compounds, and persistent organochlorides [52,53]. Findings in children (age < 14) living within 2.5 km of facilities producing/processing glass and mineral fibers included an increased risk of leukemia [53] and renal tumors [54]. Neuroblastoma in children was associated with proximity to mining industries [55]; and bone cancer was associated with proximity to cement and lime manufacturing [56]. Several other studies have examined associations with outdoor air pollution using indirect measures of exposures and report conflicting results [57,58,59].

### 3.2. Indoor Exposures

Exposures to indoor air pollution include IARC-classified carcinogens such as radon, asbestos, secondhand smoke, vehicle fuel and exhaust products, combustion products from cooking and heating and residential use of pesticides, formaldehyde found in building materials and furniture and some volatile organic compounds (VOCs) used in children’s clothing, toys, home furnishings, paint, home construction, and renovation products [60,61,62]. Direct measures include metals/substances or chemicals found in household dust, household air, and children’s urine samples. Indoor exposure to radon, which produces ionizing radiation, is discussed in Section 4.2 [62,63].

A pooled analysis of eight studies conducted as part of the Childhood Leukemia International Consortium demonstrated increased risk of ALL in children (age < 15) associated with parental exposure to home painting 1 to 3 months before conception, during pregnancy, and after birth (OR 1.54; 1.28–1.85, OR 1.14; 1.04–1.25 and OR 1.22; 1.07–1.39, respectively) [12].

Residential pesticide exposure, assessed in a meta-analysis of 15 case–control studies published 2009–2018, showed a significant association (and no evidence of publication bias) with overall childhood leukemia risk (OR 1.57; 1.27–1.95) for eight studies of ALL (pooled OR 1.42; 1.13–1.80) and for five studies of AML (OR 1.90; 1.35–2.67) [15]. The same report stratified analyses according to study quality and available exposure variables; associations with leukemia were seen for residential pesticide exposure in “higher quality” studies (OR 1.65; 1.32–2.05). Specific types of exposure associated with childhood leukemia included pet treatments (OR 1.41; 1.11–1.78), professional pest extermination (OR 1.47; 1.20–1.80), insect repellent (OR 1.38; 1.04–1.84), and treatments for mosquitoes (OR 1.88; 1.31–2.70), but not for treatments for moths or cockroaches. Another meta-analysis also found an association between residential pesticide and increased risk of childhood brain tumors (OR 1.26; 1.13–1.40) with no evidence of study heterogeneity or publication bias [16]. A meta-analysis of mostly small studies conducted before 2010 assessed childhood (age < 25) brain tumors in relation to potential risk factors, including residential pesticide use. Maternal exposure to residential pesticides during pregnancy was associated with increased risk of brain tumors in their children (RR 1.73; 1.45–2.07) with no evidence of study heterogeneity (I^2^ = 0%) [17]. Pesticide exposure during childhood was also significantly associated with brain tumor risk (RR 1.34; 1.15–1.56) yet substantial study heterogeneity was observed (I^2^ = 60%). Finally, a study using data from the U.S.–Canada Children’s Oncology Group (COG) showed a marginally significant association between residential exposure to parental insecticide use and the use of professional lawn/landscape services and unilateral retinoblastoma (99 cases, OR 2.8; 1.1–6.7 and OR 2.8; 1.0–8.2, respectively) [64]. Neither the timing of exposure related to pregnancy (before or during), nor the use of insecticides inside or outside the home modified the observed risk estimates.

Other individual case–control studies conducted within the California Leukemia Study (1995–2008) assessed the risk of leukemia from birth to diagnosis (age < 15) associated with indoor air pollutants measured through household dust sampling methods. Relationships were noted for polycyclic aromatic hydrocarbons (PAHs) [65], polybrominated diphenyl ethers [66], and from indoor home remodeling [67]. An association with leukemia was not found with any of nine metals, including copper, lead, and arsenic measured in household carpet dust [68]. The nationwide Australian Study of Childhood Brain Tumors found an association between brain tumors (age < 15) and prenatal or postnatal exposure to domestic wood heaters [51].

A hospital-based case–control study in China identified associations between ALL in children (age < 16) and maternal prenatal exposure to interior home renovations or pesticides [69]. In Shanghai, a hospital-based case–control study of children (age < 15) found higher levels of organophosphate pesticide-related dialkylphosphate metabolites in acute leukemia cases compared to controls; parental self-reported household use of mosquito repellant was associated with an approximate doubling of ALL risk [70]. A case–control study from the same group reported increased risk of acute leukemia and high indoor concentrations of VOCs [71]. A small case–control study in Shanghai showed that acute leukemia in children (age < 15) was associated with higher household air levels of nitrogen dioxide and numerous volatile organic compounds, which are pollutants attributed to synthetic building materials and household furnishings, paternal smoking, and pollution from outside traffic or industrial processes [72]. In Mexico, a case–control study (n = 195 cases and n = 369 controls) of infants (age < 24 months) found elevated risk of parental exposures to petroleum-based substances at home among infants with acute leukemia compared to controls [73].

## 4. Radiation

The association between childhood cancer and maternal exposure to ionizing radiation during pregnancy is discussed in Part 2 of this review series [2]. Ionizing radiation from medical procedures and ultraviolet (UV) radiation during childhood was summarized in Part 1 [1], and prenatal exposures from medical procedures were summarized in Part 2 [2]. Guidelines have been developed to reduce exposures to ionizing radiation during medical care and in occupational settings [74,75,76].

### 4.1. Radiation from Outdoor Sources

This section summarizes evidence relating to sources of radiation typically originating outdoors, including ionizing radiation from nuclear power plants and radon, non-ionizing radiation from electromagnetic fields (EMFs), ultraviolet radiation (UV), and occupational exposures.

#### 4.1.1. Ionizing Radiation

Due to its ability to cause cancer by damaging cells and DNA, IARC classifies ionizing radiation as a human carcinogen (Group 1) [14]. Despite this biological plausibility and elevated leukemia rates among atomic bomb survivors, evidence is mixed for an association between childhood leukemia and residential proximity to nuclear power facilities. Studies addressing the associations between childhood cancers and proximity to nuclear power facilities are often ecological in design and thus lack individual exposure and outcome measures, which, along with an absence of exposure correlates, limits confidence in study results. Numerous federal safety requirements are in place with the aim of protecting the communities near these facilities from exposure to radiation [77]. In 2010, against a backdrop of community concern but mixed evidence of childhood cancer risk [78,79,80], the U.S. Nuclear Regulatory Commission (NRC) asked the National Academy of Sciences (NAS) to study the feasibility of assessing cancer risk in populations living near nuclear power plants [30]. The NAS determined that such a study would cost tens of millions of dollars without any assurance of producing a definitive answer, and the project was not pursued [81]. The possible link between nuclear power plant radiation and cancer risk seems unlikely to be addressed by more definitive studies.

#### 4.1.2. Non-Ionizing Radiation

Both radiofrequency electromagnetic fields (RF-EMFs) and extremely low frequency (ELF) magnetic fields are classified by IARC as possibly carcinogenic to humans (Group 2B) [82,83]. This section reviews childhood cancer risk associated with low and medium EMF arising from environmental and parental occupational exposures. The studies reviewed here examine pre-conception, prenatal, and childhood exposure timeframes in relation to childhood cancer risk. Exposure to high frequency ionizing EMF from medical procedures (e.g., magnetic resonance imaging) is summarized in Part 1 [1]. Studies of parental occupational exposure to EMF are described under Occupational Exposures (Section 5.2, below).

Environmental exposure to low- and medium-energy EMF arises from a vast and diverse array of sources outdoors (including UV radiation, power lines, wireless networks, and other wireless communications and signals) or indoors (including interior electrical wiring, industrial and domestic appliances, microwaves, television and computer screens, wireless networks, and cellular and some cordless phones) [84]. In contrast to high-energy ionizing EMF, low and medium non-ionizing electromagnetic radiation lacks sufficient energy to damage cells or DNA. The mechanism through which these forms of EMF might cause cancer has not been identified.

Despite extensive study, evidence of causal associations between EMF and childhood cancer, specifically leukemia, has been elusive. Difficulties in epidemiologic study methods include exposure measurement, selection of controls, adjustment for confounders, and exposure to other carcinogens in the home. Biologically, the association has not been adequately explained mechanistically, and animal models are lacking. A study using simulations and sensitivity analyses suggested that uncontrolled confounding by residential mobility (a proxy for childhood infection) might spuriously inflate the effects of EMF exposures on childhood leukemia, but concluded the bias was not sufficient to fully account for positive findings [85]. In any case, the true impact of non-ionizing radiation on childhood cancer is uncertain, as well as the possible roles for childhood infection and population mixing (discussed in Part 1 [1]).

A review of 44 studies conducted since 1990 concluded that estimates of relative risk from exposure to EMF have declined over time; however, the decrease was not statistically significant, and the reason for the decline is not clear [86]. A meta-analysis of 12 case–control studies reported a 33% higher risk of childhood cancer in children exposed to EMF (OR 1.33; 1.10–1.60) with no evidence of statistical heterogeneity (I^2^ = 0%) or publication bias [31]. However, the authors emphasized that the results should be considered with caution because many possible confounders could not be adequately addressed with the available data. An earlier meta-analysis relating to childhood leukemia and ALL also found positive associations with EMF (OR 1.57; 1.03–2.40, and OR 2.43; 1.30–4.55, respectively) [32]. Given mixed evidence and increases in the use of personal computers, cell phones, and domestic wifi networks, potential risks associated with EMF warrant consideration in future studies.

In some case–control studies, residential proximity to power lines, underground cables, and transformer stations has served as a proxy for EMF and ELF-MF exposure. Exposures greater than 0.3–0.4 microtesla (µT) from these sources have been associated with small but consistently increased leukemia risks. The California Power Line Study, a large records-based case–control study, found a four-fold increased leukemia risk associated with proximity to high voltage lines (<50 m) combined with high calculated EMF (≥0.4 µT) [33]. A case–control study in Iran found possible associations between ALL and pre- and postnatal exposure for >4 years to high voltage lines, but interpretation may be limited by the small cohort size [87].

Several other studies have shown largely null results, including a study of EMF from high voltage transformer stations and transmission lines. A case–control study in California found no association between wire codes (a method of determining EMF exposure) and childhood leukemia but highlighted the challenges in selecting the control group [34]. A case–control study conducted in Mexico City showed a linear association between ELF-MF (five exposure levels based on magnetic flux density measured for 24 h in children’s bedrooms) and B-cell ALL [35]. After adjusting for covariates, ELF-MF exposure was associated with moderate risk of developing B-cell ALL, which is seen most clearly in children between 5 and 10 years of age [35]. Several studies found no clear evidence of risk associated with EMF [88,89,90,91,92], or specifically with radio frequency broadcast transmitters [93]. For parental occupational exposures to EMF, see Section 5.2.

### 4.2. Radiation from Indoor Sources

Radon is a natural radioactive gas that arises from underground deposits of the radioactive metals, uranium, thorium, or radium; these metals release gases into the soil, ground water, and air [94]. Contaminated air and water enter buildings through cracks in basements, sump pumps, gaps around service pipes and plumbing that draws water from radon-contaminated wells; radon and its radioactive particles enter the human body primarily through inhalation [95]. IARC classifies radon as carcinogenic to humans (Group 1) because it is a well-established risk factor for adult lung cancer, and second only to cigarette smoking as the most common cause of this disease [63].

Indoor radon levels in buildings are measured by detectors that are typically placed in basements, where radon levels are likely to be highest. The World Health Organization (WHO) defines the radon mitigation action level at 2.7 pCi/L [96], whereas the U.S. Environmental Protection Agency (EPA) action level is 4 pCi/L [63], with a recommendation to consider mitigation in homes with levels between 2 and 4 pCi/L. These recommendations balance human risks with practical issues relating to the reductions that can be realistically achieved through radon mitigation strategies which promote movement of air from the home’s foundation to the outside [95]. One in fifteen houses in North America have radon exposure levels that exceed the 4 pCi/L action level, and the proportion of affected houses is increasing over time, possibly as a result of energy-conserving measures that decrease exchange between indoor and outdoor air, resulting in build-up of radon indoors [97,98].

Meta-analyses suggest a modest association between domestic radon exposure and childhood leukemia. A meta-analysis based on eight case–control and two cohort studies of childhood leukemia produced an OR of 1.22 (1.01–1.42) for the case–control studies, most of which measured radon in individual homes, but found no association in the cohort studies, which did not use direct radon measures [27]. The authors also found no evidence of heterogeneity or publication bias. A study modeling relative risks reported by previous studies linking modifiable environmental exposures to childhood cancer, and prevalence estimates for the same modifiable environmental exposures in California, suggested that indoor radon exposure may be the largest contributor to childhood leukemia occurring before age 15 in CA [28].

Most discrepancies in individual study results probably reflect variation in methods of radon measurement. Many studies use geographical or modeled estimates of radon levels, while others measure radon in the child’s household. Even individual household measurement may not reflect the child’s exposure, as levels are influenced by ambient outdoor temperatures (radon levels decrease when doors and windows are open), whether the detector is located near the child’s usual indoor location, and how much time the child spends indoors. In addition, individual household measurement may be limited to the child’s current residence. Future studies that use personal dosimeters to directly measure individual exposure could better address the limitation of childhood associations with leukemia but cannot assess past exposures. The issue of lag time between exposure and disease is also potentially problematic, particularly when the child’s residence has changed, and only the current residence is assessed. Similar limitations would affect studies of prenatal exposure.

## 5. Occupational Exposures

Aside from occupational exposures to medical ionizing radiation addressed above, studies of parental occupational exposures to carcinogens have largely focused on benzene, which is often measured indirectly through exposure to paints or solvents. Studies and meta-analyses that combined household, environmental, and/or occupational exposure to benzene are reviewed in this section. Both maternal and paternal exposures have been reported, and exposure timeframes include pre-conception, prenatal, and childhood exposures.

### 5.1. Occupational Exposures to Benzene

As noted above, IARC classifies benzene as carcinogenic (Group 1) [18], and it is present in cigarette smoke and combustion products, including vehicular exhaust emissions. In the occupational setting, benzene exposure also occurs from paints, lacquers, solvents, plastics, lubricants, rubbers, dyes, printing, detergents, drugs, and pesticides, including the organochlorine compound pentachlorophenol (PCP), which is used as a pesticide and a disinfectant. Studies on occupational exposure to benzene are often limited by the need to estimate exposures indirectly, for example, through a job exposure matrix which estimates exposure based on a job title and industry.

Nonetheless, the results of studies assessing benzene in relation to childhood leukemia are remarkably consistent, with only a few studies that do not support this association. Variations in study results may reflect exposure misclassification that is inherent in relying upon job title or industry as proxy variables for individual exposure measures, variations in the specific type and components of proxy variables (e.g., paint), as well as confounding by unmeasured covariates. Parental exposure to benzene through work environments assessed in a meta-analysis of 13 observational studies from countries around the world was associated with an increased risk of childhood leukemia (RR 1.72; 1.28–2.31) [21]. Elevated risk of leukemia was associated with maternal and paternal benzene exposure (work and non-work exposures) in a meta-analysis of 20 studies (RR 1.96; 1.39–2.78, and RR 1.23; 1.02–1.47, respectively) [19]. In a meta-analysis of seven studies, increased risk of childhood ALL was seen with maternal work and non-work exposure to petroleum (OR 1.42; 1.10–1.84), solvents (OR 1.25; 1.09–1.45), and paint (OR 1.23; 1.02–1.47) [20]. Another meta-analysis found an overall association between childhood leukemia and combined occupational and household use of benzenes and solvents (RR 1.96; 1.53–2.52); the risk was greater for AML (RR 2.34; 1.72–3.18) than for ALL (RR 1.57; 1.21–2.05) [6]. However, a pooled analysis of 13 studies conducted as part of the Childhood Leukemia International Consortium did not find an increased risk of either ALL or AML associated with parental occupational exposure to paint [99]. Nonetheless, the synthesis of the literature provided by the reviews offers strong evidence for increased risk of childhood cancer associated with parental occupational exposure to benzene.

Individual studies provide additional evidence for parental occupational exposure to benzene and childhood leukemia risk. The Swiss National Cohort Study, a census-based study of childhood cancers (age < 16) identified through the Swiss Childhood Cancer Registry, observed an association between maternal occupational exposure to benzene and increased risk of leukemia, and specifically with ALL [22]. In this study, children who entered the 1990 or 2000 census were followed through the national cancer registry until 2008 (over 9 million childhood years of observation), estimating maternal exposure to benzene based on occupation at the time of the child’s census entry. Analyses included multivariable adjustment for demographic factors and other potential carcinogenic exposures, including non-occupational exposure to benzene, background ionizing radiation, and electromagnetic fields from radio and TV transmitters.

Case–control studies based on the Denmark Cancer Registry captured all pediatric cancer cases (age < 20) occurring over almost 50 years and linked those outcomes to parental employment histories. In a subset of childhood cancers diagnosed (1968–1974) before benzene was banned from workplaces in 1975, maternal exposure to benzene during pregnancy was associated with increased ALL; evidence for paternal exposure within 3 months before conception was inconclusive [23]. However, in a separate report based on the full cohort, leukemia risk was elevated for children whose fathers worked in either the painting or printing industry during the year before the child’s birth [13]. An increased risk of CNS tumors was observed for children whose mothers worked in the painting industry in the time period from the year preceding delivery to one year after their child’s birth. A father’s employment in printing was associated with a doubling of risk of childhood CNS tumors [13].

A case–control study in California (2000–2008) found an increased risk of ALL and paternal occupational exposure to organic solvents, which contain benzene and other carcinogens [24]. A registry-based case–control study in the UK did not find an association between childhood lymphoma and paternal occupational exposure to solvents [100]. In Mexico, a case–control study of infants (age ≤ 24 months) did not detect an association between childhood acute leukemia and paternal or maternal occupational exposures to combined carcinogens (IARC Group 1) during pre-conception, pregnancy, or after the child’s birth [73]. A nearly four-fold association was seen with metal dusts, but the confidence intervals were wide, and the case counts were too small to allow multivariable analysis.

### 5.2. Miscellaneous Occupational Exposures

The paragraphs below review individual studies of childhood cancer risks in relation to less commonly investigated occupational exposures, including diesel exhaust, EMF, ceramics/glass, various dusts, lead, and fumes. A few studies assessed childhood cancer risk in relation to any or combined exposure to diverse occupational agents. Timeframes studied included pre-conception and pregnancy exposures, and exposures occurring during the child’s lifetime.

In Denmark, a registry-based nested case–control study examined paternal occupational exposure to diesel exhaust one year prior to the child’s birth in relation to leukemia and CNS tumors in children (age < 20) [101]. Maternal exposure was assessed for the two-year period beginning a year before the child’s birth and ending a year after. Neither maternal nor paternal diesel exhaust exposure was associated with childhood leukemia. Paternal occupational exposure to diesel exhaust was unrelated to CNS tumors. However, a significant, positive association was found between maternal exposure to diesel engine exhaust and the risk of astrocytoma, and evidence suggested a higher risk for CNS tumors overall. In a separate report by the same group, maternal occupational exposure to wood dust was associated with offspring ALL and AML, paternal exposure to wood dust was associated with the risk of astrocytoma, and maternal exposure to paper dust was associated with CNS cancers [102].

Parental occupational EMF exposure and the risk of bone cancers in children (age < 15) was assessed in a population-based case–control study using five decades of data (1962–2010) from the United Kingdom National Registry of Childhood Tumours [102]. Fathers’ self-reported occupational exposure to EMF at the time of the child’s birth was associated with rhabdomyosarcoma. The study demonstrated that self-reported paternal occupational exposure to textile dust at the time of the child’s birth was associated with an increased risk for the Ewing sarcoma family of tumors. A separate report based on the same resources found significant associations between exposures to ceramics and/or glass and Hodgkin lymphoma and non-Hodgkin lymphoma, between exposure to metal fumes and Hodgkin lymphoma, and between Burkitt lymphoma and paternal occupational exposure to lead at the time of the child’s birth [100].

A small case–control study of paternal occupational exposure to non-benzene aromatic solvents in the year before conception demonstrated increased risk of brain tumors in children (age 0–14) after adjustment for parental education and occupational exposure to diesel exhaust, which contains polycyclic aromatic hydrocarbons (PAHs) (IARC Group 2A, probable carcinogen) and soot [103]. A case–control study in California (2000–2008) found a relation between ALL and paternal occupational exposure to PAH in Latino fathers [24].

A registry-based study in Denmark enrolled 144 retinoblastoma cases (age < 5) and 3600 controls [104]. No significant associations were observed for maternal occupations occurring from conception to birth; however, paternal employment in the food/drink industry (OR 2.18; 1.13–4.21) and metal/iron manufacturing (OR 1.63; 1.02–2.59) during the 90 days prior to conception was associated with an increased risk of childhood retinoblastoma after covariate adjustment [103]. The association with metal/iron work was slightly stronger for bilateral retinoblastoma. A multicenter case–control study based on data from the U.S.–Canada-based Children’s Oncology Group (COG) study assessed parental occupational exposures to several chemicals (pesticides, welding and non-welding metal fumes, sulfur dioxide, PAH, ionizing radiation, paints, and various volatile organic compounds (VOCs)) [105]. Parents of 187 children with unilateral and 95 with bilateral retinoblastoma diagnosed between 2006 and 2012 were compared to parents from 155 friend controls. In the assessment of individual exposures, the risk of bilateral retinoblastoma appeared to be elevated only for paternal paint exposure during the ten years of pre-conception (OR 8.76; 1.32–58.09), but the OR was unadjusted and the CI was very wide. The estimate for paternal exposure to any one of the many exposure possibilities did not reach statistical significance after covariate adjustment. The OR for bilateral retinoblastoma was 6.59 (1.34–32.40) with at least one exposure in fathers > 30, but again the CI was wide. In mothers, occupational exposure to at least one chemical during the timeframe starting six months before conception through delivery was associated with an increased risk of unilateral retinoblastoma (OR 5.25; 1.14–24.20), but again, confidence intervals were wide. An association was also suggested for bilateral retinoblastoma, but the CIs were also wide (56 cases, OR 3.4; 0.6–18.0) likely due to small case numbers. A separate case–control study conducted by COG found that paternal exposure to paints, after adjustment for household income, was significantly associated with increased odds of hepatoblastoma (the effect estimate for maternal exposure was larger but not statistically significant) [106].

### 5.3. Agricultural Animals

This section reviews studies assessing parents’ occupational and non-occupational as well as children’s exposure to agricultural animals. Exposure to agricultural animals has been hypothesized as a risk factor for childhood cancer primarily because of the presence of infectious agents [107]. Exposures have been assessed during the pre-conception and pregnancy timeframes, and after the child’s birth.

A systematic review of various factors in relation to childhood brain cancer did not find clear evidence of increased risk in children whose mothers lived/worked on a farm while pregnant [17]. Other ecological, population-based single studies demonstrated some evidence of increased risk of childhood CNS and AML in relation to agricultural exposures. For example, an ecological study in nine U.S. states assessed cancer cases (age < 5) at the county level in relation to county-level densities of animal inventories and animal operations [36]. No risk of childhood cancer was observed for cattle, equine, or goat densities, but broiler chicken density was associated with AML rates, and ALL rates increased with an increasing density of hog operations. However, the weakness of the ecological study design makes it impossible to infer any causal association.

A population-based, case–control study in Denmark based on linking pension records (to determine occupation) with cancer registries identified cancers occurring in children (age < 17) between 1968 and 2016 [37]. The results showed an association between CNS tumors and paternal exposure to livestock and animal dust from the child’s birth to diagnosis. Maternal pregnancy exposure was associated with a borderline increased risk of astrocytoma. In rural areas, maternal livestock and animal dust exposure from the child’s birth to diagnosis was associated with childhood AML.

Agricultural exposures were assessed by the International Childhood Cancer Cohort Consortium in a prospective study of birth cohorts from five countries (Australia, Denmark, Israel, Norway, and the United Kingdom), which included 329,658 families and identified cancers in children (age < 16) [38]. AML was significantly associated with paternal occupational exposure to animals or organic dust (although organic dust exposure in fathers was inversely associated with ALL). Low prevalence of exposures in mothers prevented meaningful analyses of risk for childhood tumors. A small case–control study conducted in Greece identified a significantly increased risk of brain tumors in children who had lived on a farm [39].

### 5.4. Agricultural Pesticides

A meta-analysis of parental occupational pesticide exposure in relation to childhood brain tumors provided summary estimates for sixteen case–control studies (summary OR 1.30; 1.11–1.50) and five cohort studies (summary RR 1.53; 1.20–1.95) [25]. In a population-based case–control study using the Spanish Cancer Registry, children’s residential proximity to cultivated agricultural regions—considered a proxy variable for pesticide exposure—was associated with excess risk of several types of childhood cancers including leukemia, non-Hodgkin lymphoma, and neuroblastoma [108]. However, a registry-based case–control study in the UK did not find an association between paternal occupational exposure to pesticides and childhood lymphoma [100].

Conflicting study results may reflect the absence of accurate individual exposure measures and use of different or suboptimal proxy variables. The accuracy of individual measures may be reduced by unknown conditions, such as, for example, the extent to which an individual used protective gear to avoid direct handling or inhalation. Conflicting views also exist regarding the carcinogenic potential of some pesticides. In 2015, an IARC panel of experts from 11 countries summarized the evidence from about 1000 studies examining cancer risk associated with five organophosphate chemicals [109]. The panel concluded that the herbicide glyphosate is probably carcinogenic to humans (Group 2A) based on published evidence including from laboratory animals. This classification is at odds with the EPA which currently states that glyphosate is unlikely to be carcinogenic to humans and that “EPA’s cancer evaluation is more robust than IARC’s evaluation” [110]. The latest EPA guidance states “there are no risks of concern to human health when glyphosate is used in accordance with its current label” [111]. Discussion of the ensuing litigation and evidence to further inform this controversy is beyond the scope of this review.

## 6. Limitations

This review summarizes evidence from English language papers published within a specific timeframe (2014 to early 2021) and therefore excludes older and more recent evidence, as well as research published in other languages. The results of studies reviewed here may have limited generalizability to non-westernized populations, where exposures, exposure levels, and risk estimates may differ. Unfortunately, studies on environmental and occupational exposures in relation to childhood cancer remain limited in global scope, primarily due to inconsistencies in, or complete absence of, exposure assessment methods and reliable cancer incidence or mortality data. Geographic variation in carcinogenic exposures and the limited capacity for adequate risk assessment is especially pronounced in low-resource communities, which contributes to incomplete understanding of the environmental and occupational factors influencing childhood cancer in these populations.

Substantial limitations in this research field include difficulties in measuring the exposures of interest, use of indirect exposure measures, biases of exposure recall, and the occurrence of overlapping and combined exposures. Because many harmful environmental exposures do not occur in isolation, investigators struggled to identify, measure, and evaluate all relevant environmental exposures affecting pediatric cancer risk. These challenges may affect not only the association under study, but also hinder the control of confounding by other carcinogens and/or social and structural determinants of health. In addition, findings for some exposures are based on ecological studies, which is a suboptimal study design that does not include individual exposure or outcome measures. In any study design, associations may also be affected by cognitive, reporting, or publication biases. Even well-designed studies are also limited by the challenges of enrolling adequate numbers of study participants and controls generalizable to all children diagnosed with cancer, and the rarity of the many types of pediatric cancer. These challenges not only make research harder to conduct but small sample sizes and exposure misclassification bias may limit statistical power leading to type II error and false negative conclusions concerning risk.

Finally, more research is needed to explore the synergistic effects of combined exposures and gene–environment interactions.

## 7. Summary of the Three-Part Series

**Figure 1:**
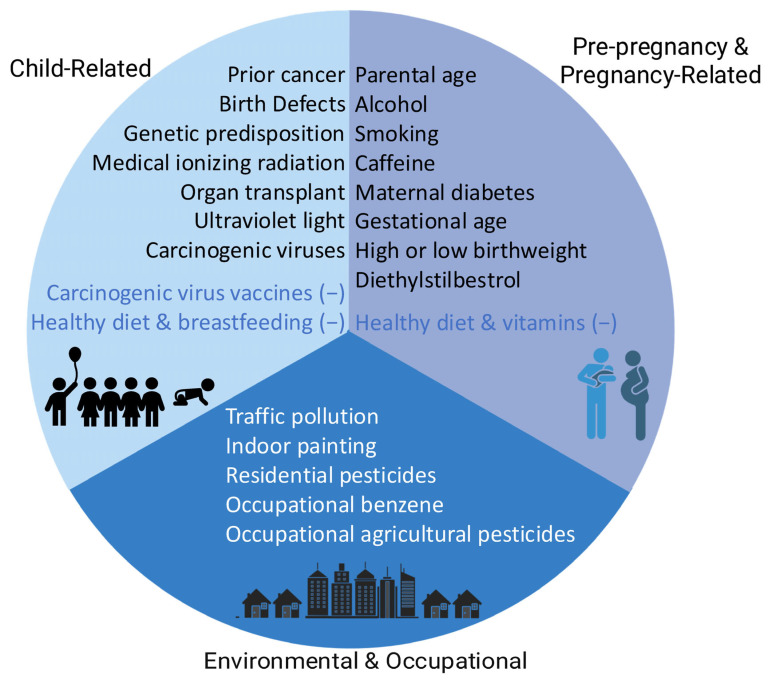
Risk factors reviewed in this series that have strong evidence of associations with childhood cancer in the literature published from 2014 to 2021 are grouped by exposure timing (pre-pregnancy, pregnancy, and child) and exposure source (environmental/occupational). Exposures in blue text are associated with reduced risk of cancer.

In this three-part narrative review series, we have summarized data from 2014 to 2021 on the etiology of childhood cancer through childhood factors [1], parental pre-pregnancy and pregnancy factors [2], and environmental and occupational factors. Many of these risk factors are currently supported by strong evidence of an association with childhood cancer risk (Figure 1, Supplementary Table S1), and of those, some are potentially modifiable. Some potential risk factors are worth addressing despite less clear evidence of a link to childhood cancer: for example, testing home air for radon and mitigating where appropriate is recommended to reduce the known risk of adult lung cancer despite uncertain risk in relation to pediatric cancer.

## 8. Conclusions

The association between childhood leukemia and exposure to outdoor traffic pollution is well documented, as is the relationship between childhood leukemia and parental occupational/nonoccupational exposure to benzene. Strong evidence also exists for leukemia and brain cancers with exposures to residential pesticides with exposure times that span pre-conception to childhood. There is compelling evidence of associations between childhood leukemia and exposure to indoor paint across all exposure periods. Strong evidence also exists for parental occupational exposure to agricultural pesticides and offspring brain cancer. We found mixed evidence for the effect of non-ionizing (EMF) and ionizing radiation exposure (domestic radon and emissions from nuclear power plants); although some studies suggest links to leukemia, interpretation of findings is compromised by exposure assessment challenges. Evidence of a relationship between exposure to agricultural animals and childhood cancer is sparse.

Future research should focus on interventions to address potentially modifiable exposures with evidence of strong associations with childhood cancer. Such exposures are related to social and structural determinants of health or occupation. Some of these harmful exposures can potentially be modified through individual and provider level interventions. Environmental and occupational exposures can potentially be modified through societal/policy level interventions.

## Figures and Tables

**Table 1:** Content of the three parts of this review covering literature published between 1 January 2014 and 17 March 2021.

Part 1 [1]. Child Factors	Part 2 [2]. Parental Pre-Pregnancy and Pregnancy Factors	Part 3 Environmental and Occupational Factors
Genetic predisposition	Alcohol	Air quality:
Birth defects	Smoking	− Outdoor exposures
Prior cancer and associated treatments	Diet and vitamins	− Indoor exposures
Medical ionizing radiation	Caffeine	Radiation:
Ultraviolet (UV) light	Parental age	− Outdoor sources
Organ transplantation	Maternal diabetes	− Ionizing radiation
Medications in childhood	Maternal obesity	− Non-ionizing radiation
Diet and breastfeeding	Birth characteristics and obstetric history:	− Indoor sources
Body mass index	− Birth weight	Occupational exposures:
Infections	− Gestational age	− Benzene
Vaccinations	− Phototherapy for jaundice	− Miscellaneous other
Allergies	− Multiple gestation	− Agricultural animals
	− Birth order	− Agricultural pesticides
	− Cesarean section and instrumental delivery	
	Assistive reproductive technologies	
	Medications during pregnancy: − Diethylstilbestrol (DES) − Other Hormones − Antibiotics and antiretrovirals − Antipyretics/analgesics − Other medications and drug use	
	Medical ionizing radiation during pregnancy	

**Table 2:** Summary of the strength of evidence for each environmental or occupational factor in relation to childhood cancer *.

Exposure	Notes
**Strong Evidence of Association with Childhood Cancer**	
Traffic pollution	The International Agency for Research on Cancer (IARC) classifies outdoor air pollution as Group 1 “carcinogenic to humans” [3]. Traffic pollution exposure to both parents and children is associated with increased childhood cancer risk. Outdoor pollution is most often associated with benzene (see below) in ambient air. Children’s residential proximity to traffic is associated with leukemia [4,5,6,7,8,9,10].
Indoor paint	IARC classifies some components of paint as a Group 1 carcinogen to humans [11]. Indoor paint exposure to parents and children is associated with leukemia [12,13].
Residential pesticides	IARC classifies several pesticides as either “probably carcinogenic to humans” (Group 2A) or “possibly carcinogenic to humans” (Group 2B) [14]. Pesticide exposure to both parents and children is associated with childhood leukemia and brain cancers [15,16,17].
Occupational/Nonoccupational Benzene	IARC classifies benzene as a human carcinogen (Group 1) [18]. Parental occupational exposure to benzene is associated with increased risk of leukemia, ALL, and AML in their children [19,20,21,22,23,24].
Agricultural pesticides	Parental occupational exposure to agricultural pesticides is associated with increased risk of brain cancer in their children [25].
**Mixed evidence (inconclusive) of association with childhood cancer**	
Radon	IARC classifies radon as a known human carcinogen (Group 1) that causes lung cancer in adults [26], but associations with childhood leukemia are mixed [27,28].
Ionizing radiation	IARC classifies ionizing radiation as a Group 1 known human carcinogen due to strong evidence for adult lung cancer [26,29]. However, evidence is mixed for an association between childhood leukemia and residential proximity to nuclear power facilities and a causal association seems difficult to prove [30].
Low and medium electromagnetic fields	IARC classifies both extremely low frequency (ELF) magnetic fields and radiofrequency electromagnetic fields (RF-EMFs) as Group 2B, which means they are “possibly carcinogenic to humans” [31,32,33,34,35]. Sources include personal computers, cell phones, and domestic wireless internet (wifi) networks. Measuring these exposures is challenging and evidence is mixed for an association with childhood leukemia.
**Weak or no evidence of association with childhood cancer**	
Agricultural animals	A systematic review found little association between childhood brain cancer and mothers who lived/worked on a farm while pregnant [17]; although a few observational studies have reported associations of parental exposures to livestock and animal dust with childhood CNS cancers and leukemia, evidence is weak [36,37,38,39].

* Strength of evidence (strong, mixed, weak/no evidence) was adapted from Lupo and Spector [40] and is described in Section 2.

## Data Availability

The search strategy is provided by Ricci et al. [1], which allows the reader to reproduce the search that identified papers for this paper.

## Supplementary Materials

The following supporting information can be downloaded at https://www.mdpi.com/article/10.3390/cancers17213516/s1, Supplementary Table S1: a. A summary of childhood cancer risk factors reviewed in this series that have strong evidence of associations with childhood cancer in literature published 2014–2019; b. A summary of childhood cancer risk factors reviewed in this series that have mixed evidence of associations with childhood cancer in literature published 2014–2019.
