# Supplementary material for: Causes of Childhood Cancer: A Literature Review (2014–2021)—Part 3: Environmental and Occupational Factors
Source: Cancers (Basel). 2025 Oct 31;17(21):3516. doi: 10.3390/cancers17213516 (PMC12609540; doi:10.3390/cancers17213516)
Supplement: Supplementary file 1 [file cancers-17-03516-s001.zip › cancers-3957282-supplementary.pdf]

Supplementary Table S1a. A summary of childhood cancer risk factors reviewed in this series that have strong evidence of associations with childhood cancer in literature published 2014-2019.

**Child factors (Ricci et al [1])**	
Genetic predisposition	Germline cancer predisposition genes are associated with increased risk of multiple childhood cancers.
Birth defects	Major birth defects and some chromosomal syndromes are associated with increased risk of multiple childhood cancers.
Prior cancer and associated treatments	Diagnosis of a first childhood cancer is associated with increased risk of a second either through predisposition genes, shared cancer risk factors, or because some cancer treatments (e.g., radiotherapy) increase subsequent cancer risk.
Medical ionizing radiation	CT scans in childhood are associated with increased risk of childhood cancer, particularly leukemia and brain cancer.
Ultraviolet light	Exposure to UV light in childhood is associated with greater risk of melanoma, usually later in life. Some states have passed legislation banning indoor tanning in minors.
Organ transplantation	Immunosuppression after solid organ transplant is associated with increased risk of several childhood cancers.
Carcinogenic viruses and vaccinations	Certain carcinogenic viruses are strongly associated with cancer in childhood and later life e.g., Epstein–Barr virus, hepatitis B virus [HBV] and human papilloma virus [HPV]. HBV vaccination is associated with lower risk of hepatocellular carcinoma. HPV vaccination appears protective against HPV-related cancers in childhood cancer survivors. It is unclear if common childhood vaccinations (against non-carcinogenic viruses) affect cancer risk.
Diet and breastfeeding	Breastfeeding is associated with a lower risk of some cancers as well as other health benefits. There was some evidence for decreased leukemia risk with better childhood diet quality. A correction has been submitted relating to Ricci et al [1] which incorrectly classified diet and breastfeeding under “mixed evidence”.
**Pre-pregnancy and pregnancy factors (Emeny et al [2])**	
Alcohol use during pregnancy	Maternal consumption of alcohol during pregnancy is associated with increased risk of several childhood cancers.
Cigarette smoking during pregnancy	Cigarette smoking in either parent, both before conception and during pregnancy, is associated with higher risk of several childhood cancers.
Diet and vitamins	Lower risk of some childhood cancers is seen with folic acid supplementation and consumption of fruits, vegetables, and legumes during pregnancy.*
Caffeine	Coffee consumption during pregnancy is associated with an increased risk of childhood leukemia.

Maternal diabetes	Pre-existing maternal diabetes is associated with an overall increased risk of offspring cancer and specifically acute lymphoblastic leukemia, but evidence is mixed regarding the role of gestational diabetes.
Gestational age	Preterm birth is associated with increased risk of several cancers. Post-term birth may be linked to increased risk of leukemia.
High or low birth weight	High or low birth weight is associated with increased risk of some childhood cancers.
Diethylstilbestrol (DES)	DES is no longer prescribed during pregnancy as it is causally associated with increased risk of cervical/vaginal clear cell adenocarcinoma in girls and young women in the first and possibly in the subsequent generation.
Parental age	Older parental age is associated with increased risk of childhood leukemia but it is difficult analyzing the associations with maternal and paternal age separately because they tend to vary together.
**Environmental or occupational factors**	
Traffic pollution	Benzene-related traffic pollution exposures in parents or children are associated with increased childhood leukemia risk. Evidence for postnatal exposure up until the time of diagnosis was strongest although a few meta-analyses demonstrated increased risk of prenatal exposure (conception or pregnancy to diagnosis) to residential proximity to high traffic density areas and gas stations.
Indoor paint	Fresh paint exposure in parents or children is associated with leukemia.
Residential pesticides	Pesticide exposure to both parents and children is associated with childhood leukemia and brain cancers.
Occupational Benzene	Parental exposure to benzene is associated with increased risk of leukemia (particularly ALL and AML), in their children.
Agricultural pesticides	Parental exposure to agricultural pesticides is associated with increased risk of brain cancer in their children.

Supplementary Table S1b. A summary of childhood cancer risk factors reviewed in this series that have mixed evidence of associations with childhood cancer in literature published 2014-2019.

**Mixed evidence**	
**Child factors (Ricci et al [1])**	
Infections	It is unclear if exposure to common childhood infections may decrease childhood cancer risk.
Allergies	Rhabdomyosarcoma is less common in children with allergies, but the evidence on associations between allergies and other childhood cancer risk is mixed. A correction has been submitted relating to Ricci et al 2024 [1] which incorrectly classified this under "strong evidence".
**Pre-pregnancy and pregnancy factors (Emeny et al [2])**	
Maternal obesity	There is mixed evidence of an association between maternal obesity and childhood cancer, after controlling for maternal diabetes and the child's birth weight.
Birth order	Later birth order may be related to decreased leukemia risk, but there is mixed evidence for other cancer types including brain tumors.
Cesarean / instrumental delivery	There is mixed evidence that Cesarean section increases total cancer risk in offspring. The risk of brain tumor may be lower in children born by Cesarean section but higher in those born by instrumental vaginal delivery. It is unclear if a causal mechanism may relate to the mode of delivery or to the indication for it.
Radiation during pregnancy	Because ionizing radiation is known to be carcinogenic, use of medical imaging in women who are pregnant or think they might be pregnant is routinely avoided where possible. This trend likely made it more difficult for recent studies to consistently find the associations reported in older studies.
**Environmental or occupational factors**	
Radon	Radon is a gas that emits ionizing radiation. It is a significant cause of lung cancer in adults but it is not clear if it causes childhood leukemia.
Ionizing radiation	Evidence is mixed for an association between childhood leukemia and residential proximity to nuclear power facilities and the impacts of exposure from nuclear power plants cannot be adequately studied.
Low and medium electromagnetic fields	Extremely low frequency (ELF) and radiofrequency electromagnetic fields are "possibly carcinogenic to humans." Sources include personal computers, cell phones, and domestic wireless internet (wifi) networks. It is challenging to prove a causal link between these exposures and childhood leukemia.
